# Pharyngeal Airway Volume Changes and Patient-Reported Breathing and Sleep Comfort Following Class II Functional Orthodontic Treatment, Predominantly the Herbst Therapy, in Growing Children: A Retrospective CBCT Study

**DOI:** 10.3390/children13070864

**Published:** 2026-06-29

**Authors:** Ersen Bilgili, Burçin Akan, Gökçenur Gökçe

**Affiliations:** 1 Department of Oral and Maxillofacial Radiology, Faculty of Dentistry, İzmir Kâtip Çelebi University, İzmir 35640, Türkiye; 2 Department of Orthodontics, Faculty of Dentistry, İzmir Kâtip Çelebi University, İzmir 35640, Türkiye; burcin.akan@ikcu.edu.tr; 3 Department of Orthodontics, Faculty of Dentistry, Marmara University, İstanbul 34854, Türkiye; gokcenur.gokce@marmara.edu.tr

**Keywords:** class II orthodontic disorder, functional orthodontic appliances, mandibular advancement, pharyngeal airway, sleep comfort, breathing comfort

## Abstract

**Objectives**: To explore volumetric changes in the nasopharynx, oropharynx, hypopharynx, and total pharyngeal airway after Class II functional orthodontic treatment and to relate these anatomical changes to patient-reported breathing and sleep comfort. **Methods**: A retrospective observational study of 63 growing patients (11–14 years) with Class II mandibular retrusion was conducted. Pre- and post-treatment cone beam computed tomography (CBCT) scans (NewTom 5G) were exported as uncompressed digital imaging and communications in medicine (DICOM) files and analyzed with Dolphin Imaging v11.9. The pharyngeal airway was manually segmented into naso-, oro-, and hypopharyngeal regions; volumes were measured twice by a single examiner to assess reliability. Inter-examiner reliability was assessed in a 15-patient subgroup. Patients completed a 5-point Likert rating of perceived breathing and sleep changes. Statistical tests included Wilcoxon signed-rank, Friedman with Holm post hoc, Mann–Whitney U, and Spearman correlation; *p* < 0.05 was considered significant. **Results**: Intra-examiner reliability was excellent (ICC1 mean 0.988), and inter-examiner reliability was good to excellent (ICC2 0.87–0.95). Mean total pharyngeal airway volume during the treatment interval in growing Class II patients treated with functional appliances increased from 18,183.62 mm^3^ at T0 to 23,524.42 mm^3^ at T1—an average augmentation of approximately 5340.80 mm^3^ (~29.4%). Regional gains followed the following pattern: Oropharynx > Nasopharynx > Hypopharynx. Nasopharyngeal and total airway changes were found to be associated with improvements in reported breathing (ρ = 0.79 and 0.69) and sleep comfort (total airway ρ = 0.74). **Conclusions**: Functional mandibular advancement in growing Class II patients was associated with enlargement of the pharyngeal airway—most notably in the oropharynx—and these volumetric gains were followed by improved patient-reported breathing and sleep comfort within the limitations of retrospective study design.

## 1. Introduction

Class II malocclusion, characterized primarily by mandibular retrusion relative to the maxilla, is among the most common problems encountered in orthodontic practice. Beyond dental misalignment, mandibular retrusion can alter tongue posture and reduce pharyngeal airway dimensions in growing children and adolescents [1]. Clinical and imaging studies have repeatedly shown that individuals with Class II skeletal patterns often present with reduced upper airway volumes or smaller cross-sectional areas, making airway assessment an essential component of treatment planning rather than an optional adjunct [2,3].

The dimensions of the pharyngeal airway are closely associated with sagittal maxillomandibular relationships, particularly the anteroposterior position of the mandible. Since mandibular retrognathia can lead to the tongue–hyoid complex being displaced posteriorly, resulting in upper airway narrowing, functional mandibular advancement therapy in growing Class II patients could be considered a clinically relevant approach to improving both sagittal skeletal relationships and pharyngeal airway dimensions [4].

Fixed orthodontic treatment involving brackets, arch wires, elastics, distalization and mechanical appliances, with or without tooth extraction, does not significantly impact mandibular growth. It primarily corrects Class II malocclusion through maxillary incisor retraction, mandibular incisor proclination, molar distalization, Class II elastics or extraction-based camouflage. Therefore, the skeletal Class II relationship may persist, with only the dental relationship and profile being improved. Therefore, fixed treatment alone does not provide true skeletal correction in adult or post-growth cases of severe mandibular retrognathia [5].

Functional appliances for Class II correction advance the mandible to correct sagittal discrepancies. This mandibular advancement also repositions adjacent soft tissues and can enlarge the pharyngeal lumen, particularly in the naso- and oropharyngeal regions [6,7,8,9]. The magnitude and distribution of airway changes, however, depend on appliance design, the patient’s growth stage, and baseline anatomy; therefore, standardized, reproducible evaluation methods are required to compare outcomes across studies [8,9].

For reproducible assessment, the pharyngeal airway is conventionally segmented into three anatomical regions: the nasopharynx (posterior nasal spine to the soft palate), the oropharynx (soft palate to the upper border of the epiglottis), and the hypopharynx (epiglottis to the laryngeal inlet). Accurate landmark placement and consistent segmentation planes are critical because small deviations can meaningfully alter volumetric results [10].

Airway volume is associated with breathing mechanics and sleep physiology. A narrowed airway is reported to influence airflow resistance, which can promote mouth breathing, snoring, and obstructive events during sleep, whether related to obesity or not [11]. Because airway narrowing is a recognized risk factor for sleep-disordered breathing, interventions that reliably expand the pharyngeal lumen may help to improve both daytime respiratory comfort and nocturnal sleep quality [12]. Conversely, airway enlargement may reduce resistance, improve airflow dynamics, and decrease the work of breathing [13].

When the airway is too narrow or collapses during sleep, the result can be sleep-disordered breathing, including obstructive sleep apnea (OSA). Patients with chronically reduced pharyngeal dimensions are more likely to sleep poorly, wake up fatigued, and experience cognitive difficulties during the day [14]. For this reason, any orthodontic or orthopedic intervention that reliably expands the airway may carry benefits that go beyond the jaws and teeth—it may improve a patient’s sleep and overall wellbeing. This broader perspective is precisely why airway evaluation deserves a routine place in orthodontic treatment planning. Pharyngeal airway volume is reported to be in association with the quality of both breathing and sleep, and some treatments or growth patterns can shift these parameters in either direction [15].

Two-dimensional cephalometry provides useful linear and area measurements but cannot capture true three-dimensional airway geometry [16]. Cone beam computed tomography (CBCT) enables volumetric analysis and minimum cross-sectional area quantification; however, accuracy depends on validated software, appropriate thresholding, and standardized segmentation protocols [17,18,19]. Together, these refinements make it possible to perform detailed, region-by-region analyses that genuinely illuminate how functional orthodontic treatment reshapes the airway.

This study set out to quantify the airway effects of Class II functional orthodontic treatment by measuring volumetric changes in the nasopharynx, oropharynx, hypopharynx, and total airway using reproducible imaging and segmentation protocols. Beyond documenting the anatomical changes, it aims to explore whether these anatomical changes are associated with patient-reported breathing and sleep comfort.

## 2. Materials and Methods

### 2.1. Patient Selection and Ethical Approval

This retrospective observational study evaluated pharyngeal airway volume changes in growing patients with Class II malocclusion treated with functional appliances. Sample size was determined with G*Power (v3.1) using an effect size of 0.5, α = 0.05, and power = 0.80; the required sample was 51, and the final cohort comprised 63 patients (39 males, 24 females), yielding an achieved power of 0.87. Eight patients received Twin Block therapy, and 55 received Herbst treatment. Due to the insufficient number of Twin Block patients, appliance-specific subgroup analyses could not be performed. Therefore, the study is designed to evaluate generally the effects of functional mandibular advancement treatment rather than evaluating the effects of the appliances in particular. Nevertheless, in this generalized design, Herbst plays a more active role.

Inclusion criteria comprised patients with Class II malocclusion with mandibular retrusion or sagittal discrepancy, normal or reduced vertical facial height, ANB > 4°, active growth phase, and clinical indication for airway evaluation during functional orthodontic treatment. Mean treatment duration was 13 months (11–14 months).

Exclusion criteria included prior orthodontic treatment, prior otolaryngological or pharyngeal surgery, complaint of adenoid hypertrophy, tonsillar hypertrophy, allergic rhinitis, craniofacial syndromes, diagnosed sleep-disordered breathing or history of ENT treatment via medical records in the institutional automated system.

This study was approved by Izmir Katip Celebi University Non-Interventional Clinical Studies Institutional Review (decision no: 0203, date: 15 April 2021). Informed consent was obtained from all participants’ legal guardians.

### 2.2. Imaging Details

No additional imaging was performed for this study. Instead, pre- and post-treatment CBCT images that had been obtained as part of routine clinical care to assess skeletal development of the maxilla and mandible, potential root resorption, soft tissue development, and the airway, nasal cavity, and maxillary sinuses were utilized. Pre-treatment (T0) and post-treatment (T1) CBCT scans were acquired using a NewTom 5G XL unit in Safebeam™ protocol with a voxel size of 0.3 mm—which provides sufficient yet relatively low spatial resolution—combined with an 18 × 16 cm FOV, dynamic 90–110 kV voltage, current as low as 1 mA, and a scan time reduced to 5.4 s via intermittent pulsed irradiation, resulting in an effective dose of ~25–35 µSv per scan and a total of ~50–70 µSv for two scans (this value would be a total of ~16–72 µSv for two exposures for panoramic, lateral and postero-anterior cephalometric images) demonstrating compliance with ALADA principles [20,21]. Images were acquired with patients in the supine position with the head in natural posture and in maximum intercuspation. Patients were instructed to breathe through the nose, avoid swallowing, keep their tongue in contact with the palate and keep their lips slightly closed. The supine position more accurately reflects the morphology of the pharyngeal airway during sleep compared with upright two-dimensional or three-dimensional imaging modalities, as it best replicates the physiological narrowing of the pharyngeal airway, whereas upright imaging may overestimate airway dimensions due to gravitational effects, potentially leading to false-negative results [22].

Datasets were exported as uncompressed DICOM files and anonymized prior to analysis.

### 2.3. Cephalometric and Airway Analysis

**Figure 1:**
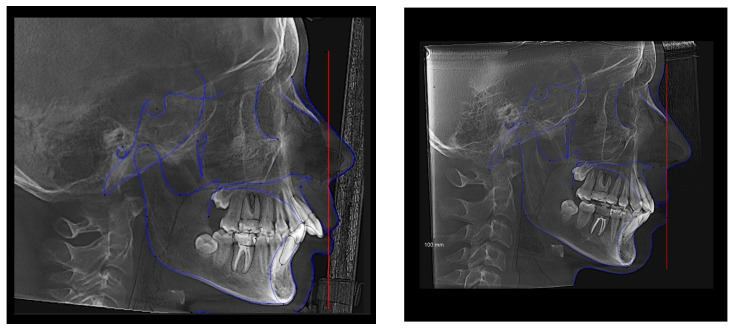
T0 and T1 cephalometric analysis from CBCT images using Dolphin software.

**Figure 2:**
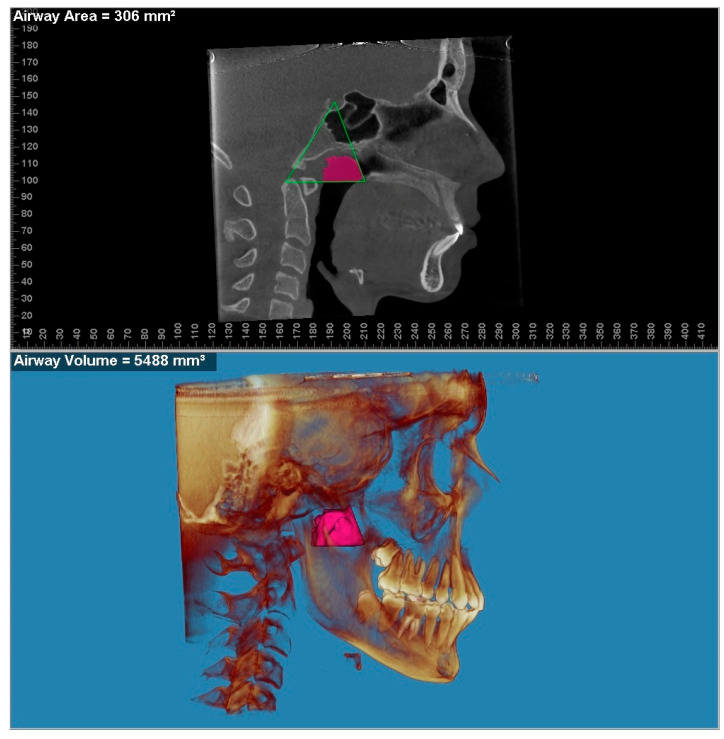
Representative nasopharyngeal airway segmentation of a single patient at T1, demonstrating area (306 mm^2^) and volume (5488 mm^3^) measurements using Dolphin Imaging software. Values reflect an individual case and do not represent the population means.

**Figure 3:**
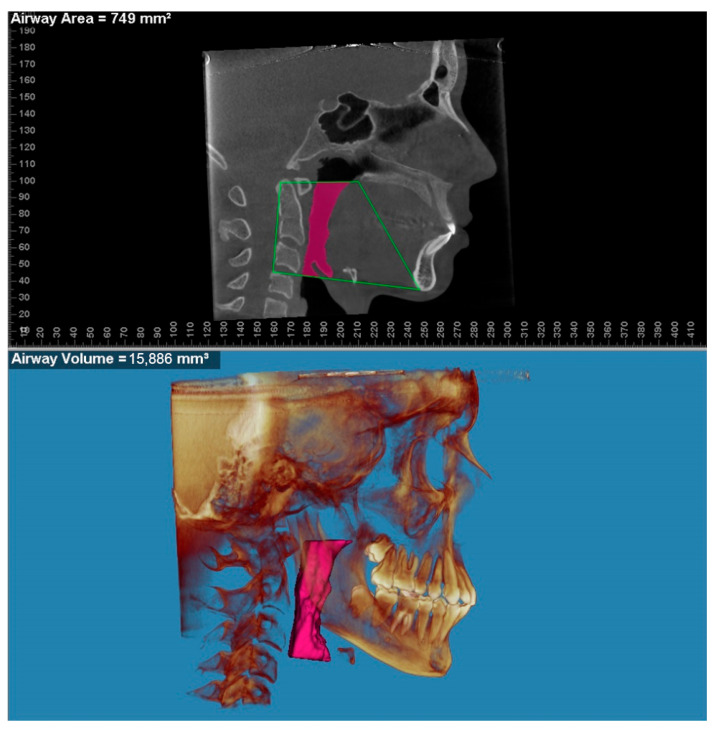
Representative oropharyngeal airway segmentation of a single patient at T1, demonstrating area (749 mm^2^) and volume (15,886 mm^3^) measurements using Dolphin Imaging software. Values reflect an individual case and do not represent the population means.

**Figure 4:**
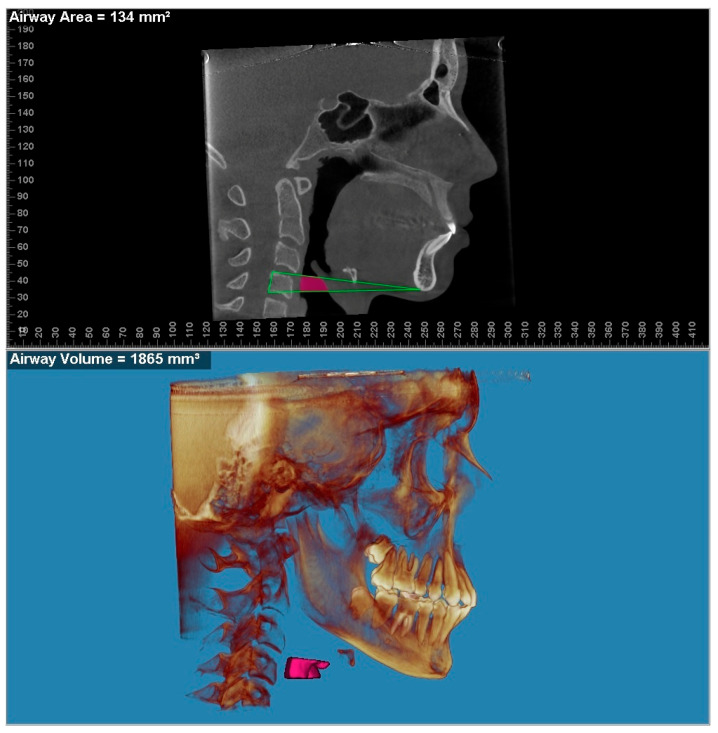
Representative hypopharyngeal airway segmentation of a single patient at T1, demonstrating area (134 mm^2^) and volume (1865 mm^3^) measurements using Dolphin Imaging software. Values reflect an individual case and do not represent the population means.

Cephalometric analysis and three-dimensional manual airway segmentation were performed using Dolphin Imaging (v11.9) (Figure 1, Figure 2, Figure 3 and Figure 4). A fixed threshold protocol was applied, with gray-level values ranging from −1000 to −587 [23].

For airway assessment, the nasopharyngeal region was defined using the center of the sella turcica, the posterior nasal spine, and the apex of the dens axis as anatomical landmarks. The oropharyngeal region was delineated superiorly by the line extending from the posterior nasal spine to the apex of the dens axis, and inferiorly/anteriorly by the menton, the base of the epiglottis, and the superior point of the C3 vertebra. The hypopharyngeal region was defined as the area bordered by the superior point of the C3 vertebra, the base of the epiglottis, the menton, and the inferior point of the C3 vertebra. Regional and total airway volumes were calculated automatically by the software’s volumetric analysis tool; while the areas determined by adhering to above mentioned anatomical landmarks are shown in green (Figure 2, Figure 3 and Figure 4).

All measurements were performed twice by the same examiner. Intra-examiner reliability was evaluated using Dahlberg’s Error Quotient, intraclass correlation coefficients (ICC), and Bland–Altman plots.

### 2.4. Patient-Reported Outcomes

While the aim was to concretely demonstrate the effects of pre- and post-treatment airway volume changes on breathing and sleep comfort, it was considered that applying the Epworth Sleepiness Scale (ESS) and the Pittsburgh Sleep Quality Index (PSQI) was deemed inappropriate, given that the participants were between 11 and 14 years of age [24,25]. Therefore, patients rated perceived changes in breathing and sleep individually on a five-point Likert scale 3 weeks after the end of the treatment (1 = negative impact; 2 = no impact; 3 = ambiguous impact; 4 = clear positive impact; 5 = strong positive impact). The same investigator, who was blinded to the volumetric airway change data, administered the questionnaire with participation from both children and their parents. And the questions posed were:

1. “Do you think your treatment, which lasted approximately one year, had an effect on your breathing comfort?”
2. “Do you think your treatment, which lasted approximately one year, had an effect on your sleep comfort?”

### 2.5. Statistical Analysis

The intra-examiner and inter-examiner reliability was assessed using Dahlberg’s Error Quotient, intra-examiner intraclass correlation coefficients (ICC1), inter-examiner intraclass correlation coefficients (ICC2) and Bland–Altman Plots. Dahlberg’s error was calculated using Microsoft Excel (Microsoft Corporation, Redmond, WA, USA) with the formula ($\sqrt{\frac{\sum d^{2}}{2n}}$) and ICCs and Bland–Altman Plots were analyzed via SPSS 21 (IBM Corp., Armonk, NY, USA).

Pretreatment and post-treatment airway volumes (T0 vs. T1) were compared for each region using Wilcoxon signed-rank tests. Inter-regional differences were assessed with the Friedman test, supplemented by post hoc Wilcoxon tests with Holm correction. Median and interquartile range (IQR) values are examined. To evaluate sex-based differences, Mann–Whitney U tests with Holm correction were applied. Spearman correlation analyses were performed to evaluate associations between regional pharyngeal airway volume changes and breathing and sleep comfort. Statistical analyses were conducted using SPSS 21 (IBM Corp., Armonk, NY, USA). ρ > 0.5 was considered indicative of a positive correlation, and *p* < 0.05 was considered indicative of statistical significance.

## 3. Results

**Figure 5:**
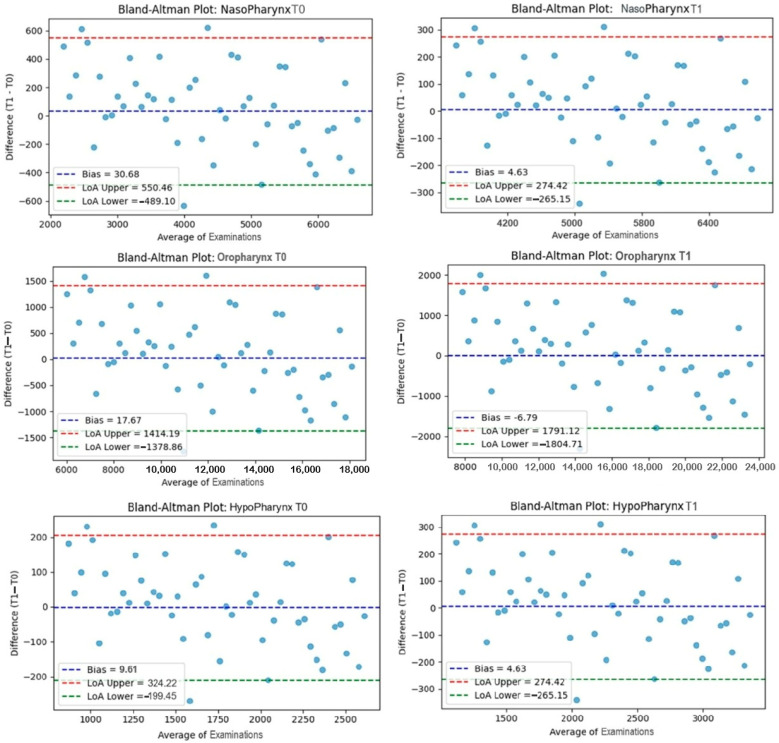
Intra-examiner Bland–Altman Plots.

This study included 63 patients who underwent functional orthodontic treatment (8 with Twin Block and 55 with Herbst appliance), with paired T0 and T1 CBCT measurements (126 scans total). Descriptive statistics reflect the predominance of the Herbst appliance in the overall results (Table 1). Intra-examiner reliability was excellent: Dahlberg errors ranged from 75.37 mm^3^ to 648.65 mm^3^ (mean 291.36 mm^3^; relative error ≈ 4.2%), Bland–Altman plots showed minimal systematic bias (mean bias +16.37 mm^3^), and ICC1 values ranged from 0.984 to 0.992 (overall mean ICC1 = 0.988), indicating near-perfect reproducibility (Table 2, Figure 5).

**Figure 6:**
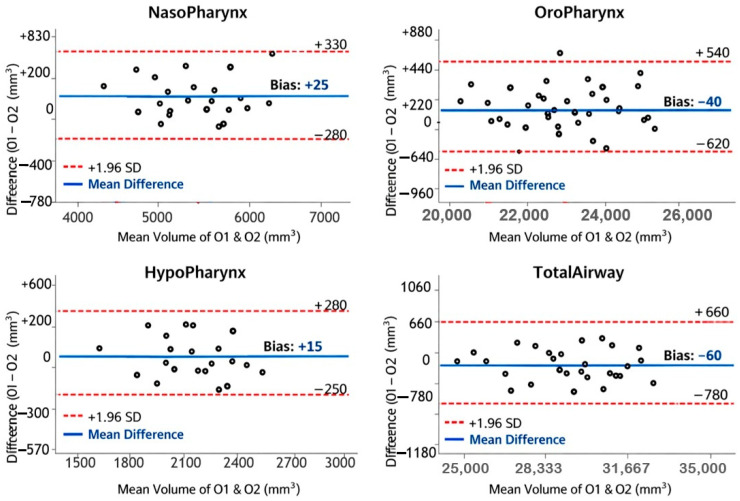
Inter-examiner Bland–Altman Plots.

To assess inter-examiner reliability, a second observer took separate airway measurements for T0 and T1 on images of 15 individuals, who were selected via an online random number generator to represent the 63 patients. The consistency of the subset consisting of eight men and seven women was analyzed using the inter-examiner Intraclass correlation coefficient (ICC2), Dahlberg error, and Bland–Altman values. Bland–Altman plots were also generated (Figure 6). The ICC2 values were between 0.87 and 0.95, the Dahlberg error was below 5%, and the Bland–Altman bias values were acceptable (Table 3).

### Volumetric Changes

Acknowledging that the study population consisted of adolescents in an active growth and development phase, mean total pharyngeal airway volume increased from 18,183.62 mm^3^ at T0 to 23,524.42 mm^3^ at T1—an average augmentation of approximately 5340.80 mm^3^ (~29.4%). Regional mean increases were greatest in the oropharynx (3627.10 mm^3^) and least in the hypopharynx (494.7 mm^3^). Paired comparisons for all nasopharynx, oropharynx, hypopharynx and total pharyngeal airway were statistically significant (Table 4).

The Friedman test indicated significant interregional differences (χ^2^(2) = 10.57, *p* = 0.005), with the hierarchy of improvement: Oropharynx > Nasopharynx > Hypopharynx (Table 5). Post hoc Wilcoxon tests with Holm correction revealed a significant pairwise difference for the oropharynx vs. hypopharynx comparison (raw *p* = 0.007; Holm-corrected *p* = 0.018; Table 6).

**Figure 7:**
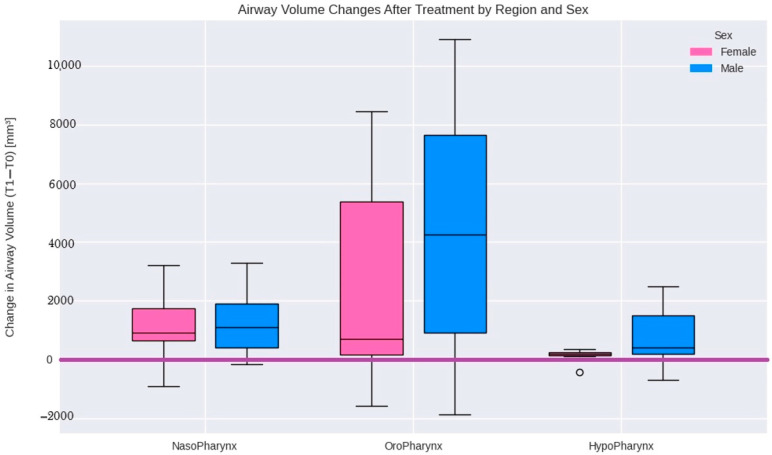
Box-Plot presentation of airway volume changes due to functional orthodontic treatment by region and sex.

Since pubertal stages and growth velocity differ between boys and girls, sex-based comparisons in the present study should be considered exploratory. Both sexes demonstrated airway enlargement, as evidenced by the findings of the present study, but males showed greater variability and slightly higher mean gains. Mann–Whitney U test results comparing volume changes (Δ) by sex lost statistical significance after Holm correction across all regions (e.g., total airway Δ: females 4660 mm^3^ vs. males 5810 mm^3^; *p* = 0.05) (Table 7, Figure 7). After Holm correction, this borderline *p*-value raises uncertainty regarding the clinical significance of sex-based airway differences.

Spearman correlations with Holm correction demonstrated positive associations between volumetric gains and perceived breathing and sleep improvements:

- Breathing comfort: nasopharynx Δ (ρ = 0.79, *p* = 0.025), total pharynx Δ (ρ = 0.69, *p* = 0.03). Oropharynx Δ (ρ = 0.55, *p* = 0.05) and hypopharynx Δ (ρ = 0.42, *p* = 0.08) did not reach significance for breathing comfort.
- Sleep comfort: total airway Δ (ρ = 0.74, *p* = 0.045), oropharynx Δ (ρ = 0.64, *p* = 0.074), nasopharynx Δ (ρ = 0.54, *p* = 0.061), hypopharynx Δ (ρ = 0.52, *p* = 0.72).

After Holm correction for Spearman, borderline *p*-values lost their statistical significance.

**Figure 8:**
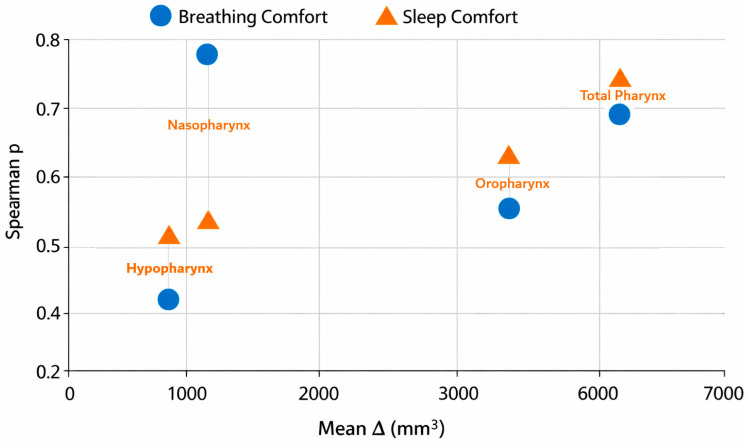
Scatter-plot displaying Spearman correlation coefficients (ρ) between regional pharyngeal airway volume changes and patient-reported breathing (blue) and sleep comfort (orange).

These results indicate that nasopharyngeal expansion was most strongly associated with patient-reported breathing comfort, while total airway enlargement was more strongly correlated with improved sleep comfort (Table 8). The scatter-plot illustrates the Spearman correlation coefficients between pharyngeal volume changes and patient-reported breathing and sleep comfort (Figure 8).

## 4. Discussion

Using CBCT and validated segmentation protocols, this study found that functional Class II treatment with Twin Block and Herbst appliances may support the enlargement of the pharyngeal airway in growing patients [6,7,8,9,15].

The reliability of the measurements was confirmed: Dahlberg’s Error and ICC values were consistent with the findings of previous studies demonstrating the excellent reproducibility of CBCT airway measurements [17,19,26]. However, CBCT analysis is influenced by patient posture, tongue position, and breathing phase. Systematic reviews underscore the necessity of standardized segmentation planes and thresholding protocols to minimize variability; furthermore, the supine imaging position, combined with other standardization measures, may more accurately reflect airway dimensions during sleep [10,16,27]. Emerging approaches, including AI-assisted segmentation, have the potential to further enhance consistency [28].

The results of the present study demonstrated a ~5340 mm^3^ increase in total pharyngeal airway volume after the implementation of a functional treatment, with the oropharynx exhibiting the most statistically significant increase, followed by the nasopharynx and hypopharynx. It is important to note that the group in which airway measurements were taken consisted of adolescents in the growth and development phase. In a study by Chan et al., the time-dependent average total pharyngeal airway change between the 9–10-year-old and 11–13-year-old groups was reported to be ~2700 mm^3^. This study involved adolescents in the growth and development phase who were not undergoing any orthodontic treatment and exhibited different skeletal patterns [29].

Consistent with the present findings, Inchingolo et al. reported significant volumetric increases in the naso- and oropharyngeal segments after Twin Block therapy [7]. Moreira Das Neves et al. found that both Twin Block and Herbst appliances resulted in increased airway dimensions. Specifically, Herbst was found to yield more substantial oropharyngeal gains [8]. Perillo et al. provided confirmation of consistent airway enlargement in Twin Block but no significant changes in Herbst treatment in their systematic review [3]. Bock et al. observed posterior airway changes during Herbst treatment, but noted variability and partial relapse after appliance removal [9]. Ahmed et al. reported airway enlargement with the Fränkel II appliance, but changes were less pronounced in the hypopharynx [30].

The hierarchy of improvement (Oropharynx Δ > Nasopharynx Δ > Hypopharynx Δ) observed in this study aligns with the findings reported by Inchingolo and Moreira Das Neves [7,8]. However, this hierarchy contrasts with reports indicating minimal nasopharyngeal changes [3,30,31,32]. Such variations can be attributed to factors such as appliance design, patient age, and segmentation protocols.

A sex-stratified analysis revealed that both males and females exhibited benefits, though males demonstrated greater variability. This result is consistent with previous studies reporting larger but more variable airway dimensions in males [26,30,31]. Vejwarakul et al. reported sex-related disparities in airway changes following orthodontic interventions, with potential implications for respiratory comfort and sleep quality [31]. Chan reported no sex differences in the improvement in the pharyngeal airway [29]. After Holm correction, the sex-based differences in the present study fell below the threshold of statistical significance, casting doubt on the clinical significance of sex-related airway differences.

Airway enlargement has been demonstrated to reduce airflow resistance, enhance breathing comfort, and potentially improve sleep quality [11,13,33]. As demonstrated in the studies by Punjabi, Chuang, and Bock, narrow pharyngeal airways have been found to be associated with sleep-disordered breathing [9,11,13]. Consequently, functional orthodontic treatment has the potential to contribute to more than just dento-skeletal correction; it can also enhance respiratory function and improve sleep health.

This study demonstrated that functional orthodontic treatment induces volumetric changes in the pharyngeal airway, with distinct segment-specific effects on patient-reported breathing and sleep comfort. Correlation analysis revealed that nasopharyngeal expansion was most strongly associated with breathing comfort (ρ = 0.79), underscoring the importance of upper airway patency in daytime respiration. In addition, total airway enlargement showed the strongest correlation with sleep comfort (ρ = 0.74), highlighting the relevance of global airway expansion for nocturnal respiration.

Segment-specific contributions were also evident: oropharyngeal changes correlated moderately with breathing comfort (ρ = 0.55) and more strongly with sleep comfort (ρ = 0.64), suggesting that mid-pharyngeal airway remodeling contributes to both domains. Hypopharyngeal changes demonstrated weaker associations (ρ = 0.42 for breathing, ρ = 0.52 for sleep), indicating a more limited role compared to other segments.

These findings align with previous literature emphasizing the clinical relevance of airway remodeling. Kırıştıoğlu et al. reported that posterior airway enlargement after orthognathic surgery reduced the risk of obstructive sleep apnea [34]. Vejwarakul et al. demonstrated that orthodontic extraction treatment altered pharyngeal airway space and was associated with changes in subjective sleep quality [31]. Savoldi et al. highlighted CBCT’s utility in airway analysis for sleep-disordered breathing [18]. A systematic review confirmed that posterior airway compromise after orthognathic surgery significantly affected sleep quality [19].

The present results corroborate these findings by demonstrating that nasopharyngeal expansion is most strongly associated with reported breathing comfort, while total airway enlargement is most strongly associated with reported sleep comfort. The integration of CBCT volumetry with patient-reported outcomes provides a comprehensive framework for evaluating treatment efficacy beyond skeletal and dental corrections.

Although the present retrospective study was designed to evaluate the general effects of functional mandibular advancement treatment rather than to compare specific appliances, the results seemed to be consistent with the existing literature.

There are several limitations of the present study. One such limitation is that, although patient medical records in the institutional automated system were reviewed to exclude medical conditions or clinical histories that may contribute to airway abnormalities, data regarding obesity status and chronic mouth breathing were not available.

Consistent with its retrospective design, additional limitations included the absence of objective sleep testing, lack of ENT assessment, and potential recall and expectation bias in patient-reported outcomes.

Despite the power analysis, the present study had a limited sample size. The lack of separate Twin Block and Herbst subgroup analyses is the main limitation of this study. The 8 patients who underwent Twin Block therapy were insufficient in number for a separate subgroup statistical analysis. Consequently, functional Class II treatment in this study was predominantly represented by the Herbst appliance, which is naturally reflected in the findings. Another limitation of this study is that in growing children, regional airway volumes naturally improve without any orthodontic intervention. However, to account for this natural growth effect, it was not possible to identify a pediatric control group with two CBCT scans obtained 13 months apart in the absence of functional orthodontic treatment. The other limitation was the method used for patient-reported outcomes in sleep and breathing comfort. Although the Epworth Sleepiness Scale (ESS) and the Pittsburgh Sleep Quality Index (PSQI) are not validated for children aged 11–14 years, a five-point Likert scale is not a validated psychometric instrument either. Therefore, the correlations reported herein should be interpreted as subjective perceptual data rather than objective outcome measures.

To this end, longitudinal studies are necessary to assess the durability of airway improvements following the removal of the appliance. Future research should integrate functional outcomes (e.g., polysomnography, sleep questionnaires) with volumetric analysis to establish stronger correlations between anatomical changes and clinical improvements.

## 5. Conclusions

Within the limitations of this retrospective, uncontrolled study, there were significant increases in pharyngeal airway volumes in growing Class II patients treated with functional mandibular advancement appliances, especially in the oropharyngeal region. These changes were found to be associated with improvements in breathing and sleep comfort as perceived by the patients. It is important to state that pharyngeal airway volumes provide valuable anatomical information but do not reflect airflow, oxygen saturation, habitual respiration patterns, or sleep-related factors. Further prospective controlled studies using validated pediatric sleep questionnaires and objective respiratory assessments are required to confirm these findings.

## Figures and Tables

**Table 1:** Descriptive statistics, including information about Twin Block and Herbst appliances, represent the predominance of the Herbst appliance (mean airway volumes at T0–T1 and changes in percentile).

Region	*n* (TW)	Twin Block (mm^3^)	T1–T0 %	*n* (Herbst)	Herbst (mm^3^)	T1–T0 %	*n* (Functional)	Functional (mm^3^)	T1–T0 %
NasopharynxT0	8	3311.63	44.50	55	4546.68	25.99	63	4389.85	27.76
OropharynxT0	8	10,409.88	36.89	55	12,285.13	29.34	63	12,051.47	30.09
HypopharynxT0	8	1503.25	36.67	55	1777.07	30.22	63	1742.30	28.40
TotalpharynxT0	8	15,224.36	36.11	55	18,614.06	28.60	63	18,183.62	29.37
NasopharynxT1	8	4785.25		55	5728.45		63	5608.68	
OropharynxT1	8	14,229.13		55	15,889.40		63	15,678.57	
HypopharynxT1	8	1708.63		55	2314.05		63	2237.17	
TotalpharynxT1	8	20,723		55	23,931.9		63	23,524.42	

**Table 2:** Intra-examiner reliability metrics (Dahlberg’s Error, Bland–Altman bias and limits of agreement, and ICC1) for each pharyngeal region.

Region	Dahlberg Error (mm^3^)	Relative Error (%)	Bias (mm^3^)	LoA Lower (mm^3^)	LoA Upper (mm^3^)	ICC1
NasopharynxT0	188.77	4.30	30.68	−489.10	550.46	0.991
OropharynxT0	503.98	4.18	17.67	−1378.86	1414.19	0.984
HypopharynxT0	199.42	4.12	9.61	−199.45	324.22	0.986
TotalpharynxT0						
NasopharynxT1	75.37	4.33	−2.78	−211.63	206.08	0.992
OropharynxT1	648.65	4.14	−6.79	−1804.71	1791.12	0.984
HypopharynxT1	97.39	4.35	4.63	−265.15	274.42	0.991
TotalpharynxT1						
Average	291.36	4.24	16.37	−789.04	821.78	0.988

**Table 3:** The ICC2 values were between 0.87 and 0.95, the Dahlberg error was below 5%, and the Bland–Altman bias values were acceptable.

Region	T0 ICC2	T1 ICC2	ICC2	T0 Error (mm^3^)	T1 Error (mm^3^)	Dahlberg	Bias	LoA (mm^3^)
Nasopharynx	0.91	0.93	Excellent	145	160	4.31%	+25	−780, +830
Oropharynx	0.89	0.92	Excellent	310	295	3.09%	−40	−960, +880
Hypopharynx	0.87	0.90	Good–Excellent	120	135	4.94%	+15	−570, +600
Totalpharynx	0.94	0.95	Excellent	420	440	3.02%	−60	−1180, +1060

**Table 4:** Wilcoxon signed-rank tests for T0 vs. T1 paired differences.

Wilcoxon SRT	Median Δ (T1–T0)	IQR (Q1–Q3)	Z-Value	*p*-Value	Effect Size (r)	95% CI for Δ
Nasopharynx	+1180 mm^3^	820–1640	−0.92	0.036 *	0.12 (small)	[+720, +1640]
Oropharynx	+3950 mm^3^	2700–5420	−3.68	0.001 *	0.46 (medium–large)	[+2800, +5100]
Hypopharynx	+970 mm^3^	620–1380	−2.11	0.034 *	0.27 (small–medium)	[+580, +1360]
Totalpharynx	+6100 mm^3^	4200–8050	−4.02	0.001 *	0.50 (large)	[+4500, +7700]

* *p* < 0.05 refers to statistical significance.

**Table 5:** Friedman test for inter-regional comparisons.

Friedman Test	χ^2^ (df = 2)	*p*-Value	Kendall’s W (Effect Size)
Naso vs. Oro vs. Hypo	10.57	0.005 *	0.18 (small–medium)

* *p* < 0.05 refers to statistical significance.

**Table 6:** Post hoc Wilcoxon tests with Holm correction.

Wilcoxon Post Hoc	Median Δ Difference	IQR	Z-Value	Raw *p*-Value	Holm- Adj. p	Effect Size (r)	95% CI
Naso vs. Oro	−2770 mm^3^	−3900–−1640	−2.14	0.032	0.05	0.28 (small–medium)	[−800, −1700]
Naso vs. Hypo	+210 mm^3^	−180–620	2.13	0.032	0.055	0.25 (small)	[−170, +610]
Oro vs. Hypo	+2980 mm^3^	+1820–+4210	2.72	0.007	0.018 *	0.35 (medium)	[+1900, +4500]

* *p* < 0.05 refers to statistical significance.

**Table 7:** Mann–Whitney U tests for sex-based Δ volume comparisons with Holm correction.

Mann- Whitney U	Median Δ (Female)	IQR (Female)	Median Δ (Male)	IQR (Male)	U Val.	*p* Val.	Holm-Corrected p	Effect Size (r)	95% CI for Δ Difference
Nasopharynx	+1050 mm^3^	880–1720	+1420 mm^3^	790–1540	468.0	0.041	0.08	0.09 (small)	[−220, +420]
Oropharynx	+3200 mm^3^	2950–5600	+3800 mm^3^	2600–5200	452.5	0.033	0.07	0.12 (small)	[−310, +710]
Hypopharynx	+410 mm^3^	250–1040	+590 mm^3^	310–1280	388.0	0.048	0.099	0.21 (sml-med)	[−90, +600]
Totalpharynx	+4660 mm^3^	3600–7400	+5810 mm^3^	4100–7900	463.0	0.029	0.05	0.10 (small)	[−480, +880]

**Table 8:** Relationship between regional pharyngeal airway volumes and comfort of breathing and sleep.

Spearman Correlation	Mean Δ (mm^3^)	Median Δ (mm^3^)	Breathing Comfort Δ		Holm Correction	Sleep Comfort Δ		Holm Correction
			ρ *	*p*	*p*	ρ *	*p*	*p*
Nasopharynx Δ	+1210	+1180	0.79 *	0.001	0.025 **	0.54 *	0.04	0.061
Oropharynx Δ	+3980	+3950	0.55 *	0.01	0.05	0.64 *	0.03	0.074
Hypopharynx Δ	+960	+970	0.42	0.08	0.24	0.52 *	0.05	0.72
Totalpharynx Δ	+6150	+6100	0.69 *	0.001	0.03 **	0.74 *	0.01	0.045 **

* ρ > 0.5 refers to positive correlation; ** *p* < 0.05 refers to statistical significance.

## Data Availability

The raw data supporting the conclusions of this article will be made available by the authors on request.

## Abbreviations

The following abbreviations are used in this manuscript:

CBCT	Cone Beam Computed Tomography
DICOM	Digital Imaging and Communications in Medicine
OSA	Obstructive Sleep Apnea
ICC1	Intra-examiner Intraclass Correlation Coefficient
ICC2	Inter-examiner Intraclass Correlation Coefficient
LoA	Limits of Agreement
IQR	Interquartile Range
ESS	Epworth Sleepiness Scale
PSQI	Pittsburgh Sleep Quality Index
